# Channels and Features Identification: A Review and a Machine-Learning Based Model With Large Scale Feature Extraction for Emotions and ASD Classification

**DOI:** 10.3389/fnins.2022.844851

**Published:** 2022-07-22

**Authors:** Abdul Rehman Aslam, Nauman Hafeez, Hadi Heidari, Muhammad Awais Bin Altaf

**Affiliations:** ^1^Department of Electrical Engineering, Lahore University of Management Sciences, Lahore, Pakistan; ^2^James Watt School of Engineering, University of Glasgow, Glasgow, United Kingdom; ^3^Department of Computer Engineering, University of Engineering and Technology-Taxila, Taxila, Pakistan; ^4^Institute of Environment, Health and Societies, Brunel University, London, United Kingdom

**Keywords:** Autism, classification processor, deep neural network (DNN), electroencephalogram (EEG), emotion detection, neurological disorder, large scale feature extraction

## Abstract

Autism Spectrum Disorder (ASD) is characterized by impairments in social and cognitive skills, emotional disorders, anxiety, and depression. The prolonged conventional ASD diagnosis raises the sheer need for early meaningful intervention. Recently different works have proposed potential for ASD diagnosis and intervention through emotions prediction using deep neural networks (DNN) and machine learning algorithms. However, these systems lack an extensive large-scale feature extraction (LSFE) analysis through multiple benchmark data sets. LSFE analysis is required to identify and utilize the most relevant features and channels for emotion recognition and ASD prediction. Considering these challenges, for the first time, we have analyzed and evaluated an extensive feature set to select the optimal features using LSFE and feature selection algorithms (FSA). A set of up to eight most suitable channels was identified using different best-case FSA. The subject-wise importance of channels and features is also identified. The proposed method provides the best-case accuracies, precision, and recall of 95, 92, and 90%, respectively, for emotions prediction using a linear support vector machine (LSVM) classifier. It also provides the best-case accuracy, precision, and recall of 100% for ASD classification. This work utilized the largest number of benchmark data sets (5) and subjects (99) for validation reported till now in the literature. The LSVM classification algorithm proposed and utilized in this work has significantly lower complexity than the DNN, convolutional neural network (CNN), Naïve Bayes, and dynamic graph CNN used in recent ASD and emotion prediction systems.

## 1. Introduction

Autism spectrum disorder (ASD) is a cognitive neurological disorder with a broad spectrum of neurological disorders characterized by social and cognitive skill impairments and physical disabilities. The physical disabilities include motor deficits, speaking and listening disabilities, inability to sit, stand, and visual impairments (Matson et al., 2011). The cognitive impairments include deficits in attention skills related to social communication and intelligence quotient (Matson et al., 2011). Another major exertion being faced by ASD patients due to physical and cognitive disabilities is emotional disorders, volatile emotions, and emotional dysregulation (Samson et al., 2014).

Emotion deregulation is described as the failure to regulate emotions appropriately and effectively (Samson et al., 2014). Maladaptive emotional responses are linked to emotion deregulation which could lead to anger control problems, temper tantrums, and aggression (Kassinove and Sukhodolsky, 1995). The emotional deregulation and maladaptive emotions cause impatience and quick anger. Quick anger can cause physical damage to self and others (Kassinove and Sukhodolsky, 1995). The early recognition of the anger triggers is important to avoid maladaptive and deregulated emotions. Therefore, it is vital to predict the emotions of ASD patients for rehabilitation (Samson et al., 2014). The recent reports on these emotional disorders for ASD patients suggest a dire need to analyze and control the emotions of ASD patients (Ghaziuddin et al., 2002).

**Figure 1:**
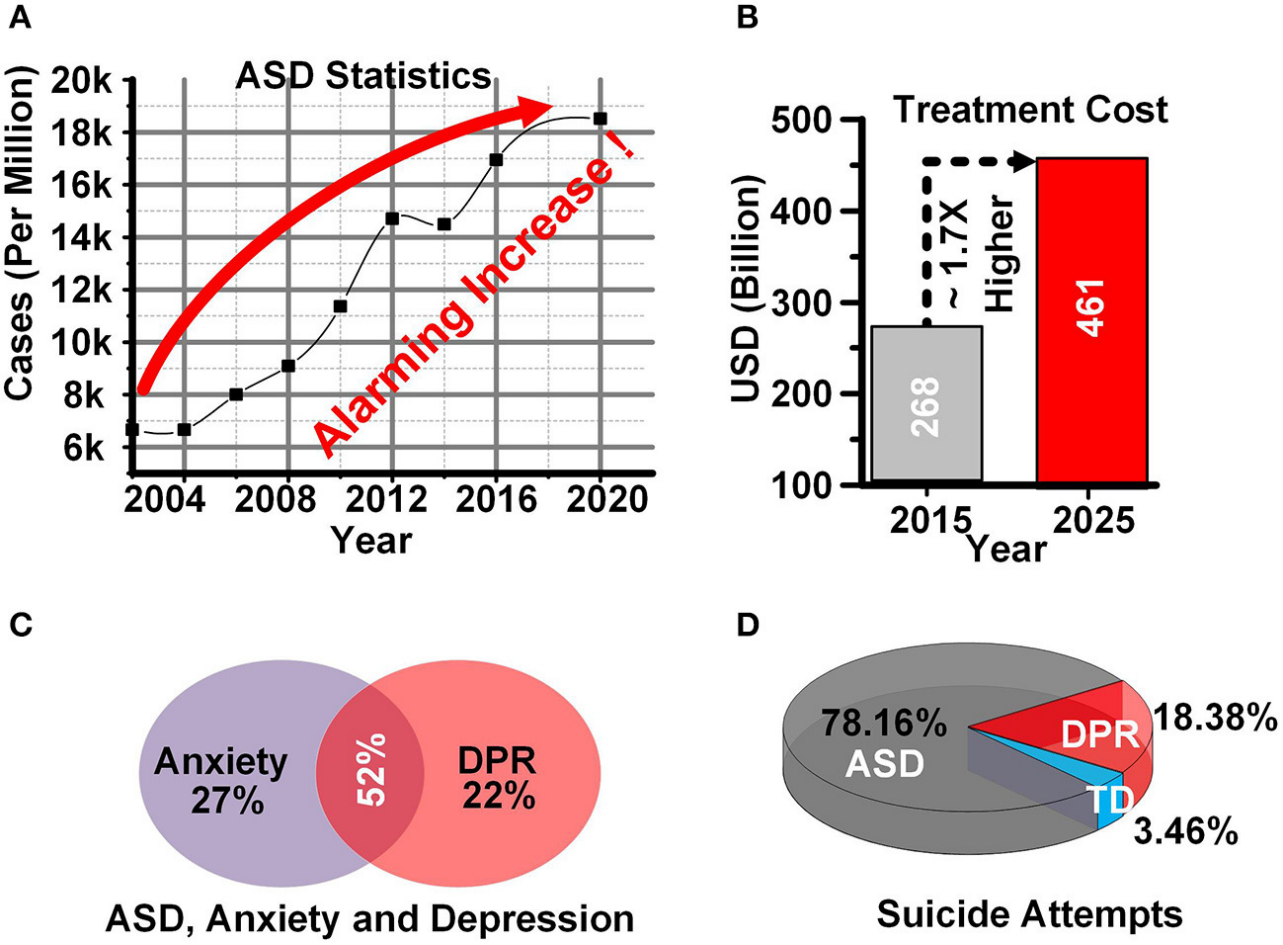
**(A)** ASD statistics **(B)** ASD treatment cost **(C)** ASD, anxiety and depression **(D)** Suicide attempts in ASD.

Figure 1 depicts some of the statistics and difficulties related to ASD patients. The Center for disease control and prevention has revealed an alarming rate of increase in ASD cases in the last 15 years and the number of cases is almost tripled among US citizens (Aslam and Altaf, 2021b). Figure 1A depicts the ratio of ASD cases per one million persons from 2004 to 2020. The economic impact of ASD is also huge. The cost of treatment for ASD children is estimated to reach 461 billion USD in 2025, which is 1.7 times higher than 268 billion (ScienceDaily, 2015) USD in 2015 as shown in Figure 1B.

ASD patients also suffer from some major issues and disorders including attention deficit hyperactivity disorder, self-injuries, bullying, anxiety, and depression (Gibbs et al., 2019). ASD patients are either affected by anxiety or depression (DPR), or by both (Figure 1C) (Aslam and Altaf, 2021b). These negative emotions constitute a significant reason for the high ratio of self-injury and suicide attempts among ASD children. Figure 1D shows the suicide attempts in ASD, typically developing (TD), and DPR children.

This high ratio of suicide attempts and ideations among ASD and depressive children is frightening and requires dire attention. Previous research has proven that an early ASD diagnosis is significantly important for the rehabilitation of ASD children (Gibbs et al., 2019). The ASD diagnosis is presently performed by an extensive set of frequent behavioral observations using the Autism diagnostic observation schedule, 2nd Edition (ADOS-2) schedule (Samson et al., 2014). A neurologist analyzes different cognitive scores of the patients with a cut-off table to identify them as ASD or TD in the ADOS-2 schedule (Samson et al., 2014). These evaluations require ample time and are a major reason for the late diagnosis of ASD patients. Therefore, the latest research focuses on the solutions to identify ASD patients using their physiological signals, especially brain signals (Electroencephalogram) (Kakkar, 2019; Alturki et al., 2021; Wadhera, 2021; Wadhera and Kakkar, 2021).

Similarly, emotion recognition and intervention can significantly aid the ASD children's rehabilitation and avoid self-injuries. Therefore, the researchers have focused on the prediction of emotions using electroencephalogram (EEG) signals (Aslam et al., 2020).

The previous works in ASD or emotions classification using EEG have provided excellent classification results using customized feature extraction and machine learning (ML) classification (Haputhanthri et al., 2019; Gonzalez et al., 2021). However, none of these works has provided 1) an in-depth analysis using large-scale feature extraction (LSFE) and feature selection algorithms (FSAs) 2) The ranking and the significance of each EEG channel for emotions and ASD classification is also missing. These investigations would be extremely beneficial for the researchers working on the development of hardware accelerators or on-chip solutions for the ASD or emotions classification (Fang et al., 2019; Aslam et al., 2021). Therefore, an in-depth analysis of the emotions and ASD classification using LSFE and different FSAs is performed to identify the most suitable channels and features.

The rest of the paper is organized as follows. The previous contributions in emotions classification and ASD prediction are discussed in Section 2. An overview of emotions and ASD classification framework is provided in Section 3. LSFE methodology is discussed in Section 4. The classification results are presented in Section 5. Finally, the discussion and conclusion of this work are presented in Section 6 and Section 7 respectively.

## 2. Previous Contributions

The state-of-the-art works for the ASD patients to regulate their emotions and an ASD diagnosis is broadly categorized into two categories a) ASD, and b) emotions classification. An emotion or ASD classification system may be providing entirely accurate (100%) classification results on a few subjects or a single data set due to over-fitting. Therefore, it is imperative to validate the system on multiple data sets and a large number of subjects. We have validated our algorithm on maximum (99) number of subjects against the previous works. The 99 subjects include 70 and 29 subjects for emotions and ASD classification, respectively. The emotions classification subjects include the subjects for three different data sets. In contrast, the ASD classification subjects include the subjects for two different data sets utilized in this work. The number of data sets is evaluated for each work based on the different data sets on which that work is evaluated. The number of data sets is an important parameter to avoid the over-fitting of ML algorithms on limited data sets.

### 2.1. ASD Classification

The ASD classification at an early age is challenging as it is conventionally diagnosed by developmental monitoring using the ADOS2 schedule. ADOS2 is the standard method for ASD diagnosis and is not fully structured (Adamou et al., 2021). It requires multiple observations and visits for a patient which are extremely time-consuming. Therefore, the average ASD diagnosis age is ≥3 years, reducing the chances for early intervention and treatment (van't Hof et al., 2021).

A significant amount of research is being carried out for early ASD diagnosis through brain activity analysis using EEG. The EEG-based ASD diagnosis would also help in mitigating the sufferings of parents or caregivers of autistic children using fully integrated wearable devices (Aslam et al., 2021).

The ASD diagnosis using EEG is quite challenging due to the limited ASD data sets and the unavailability of unified biomarkers including suitable channels, number of features, and classification algorithms. These limitations have been challenged by different researchers with excellent (> 90%) classification results. Nevertheless, none has performed the LSFE and detailed analysis to identify the best suitable channels and features for the classification. These systems are also limited to a single data set validation.

A hybrid lightweight deep feature extractor was utilized for the ASD prediction (Baygin et al., 2021). The complex deep neural networks including ShuffleNet and SqueezeNet models were utilized for the feature extraction. They have utilized a large number (32) of EEG channels. They have also focused only on the ASD classification based on a single data set. The ASD classification was performed with 96.4% accuracy using a cubic SVM classifier. The cubic SVM classifier has a higher complexity than the linear SVM classifier (Abdiansah and Wardoyo, 2015).

A three-layer neural network utilizing 63 s-order difference plot area of 64 electrodes as features with sigmoid activation function was used for ASD prediction (Abdulhay et al., 2020). The system was tested using 120 children including 60 ASD and 60 TD children and provided 94.4% classification results. Although the system provided excellent classification results for a wide range of ASD patients. But, the system is validated on a single data set, utilizes a large number (64) of electrodes, and does not provide any suitable channel and feature selection analysis.

The discrete wavelet transform was used to classify ASD with 93% classification accuracy (Haputhanthri et al., 2019). The system was tested and validated on 15 participants including 10 ASD and 5 TD persons on ODU data set. They used the discrete wavelet transform coefficients of five EEG channels and achieved maximum classification results using a random forest classification algorithm. Although they performed channel selection using a feature selection algorithm. But, the selection was handcrafted and no LSFE was performed. The system was also validated on a single data set.

A recurrent neural networks (RNN) based classifier was utilized for ASD classification using infinite impulse response filters (IIR's) (Bouallegue et al., 2020). The IIR's are generally avoided for hardware implementation due to their challenges in ensuring stability. The system was validated using KAU data set. The system provided excellent (99%) classification results. But, it also has the issue of single data set validity, utilization of a large number (16) of channels, and lack of LSFE for feature and channel selection (Bouallegue et al., 2020). This algorithm is also validated on a single ASD data set.

Similarly, another work classified ASD along with epilepsy using King Abdul Aziz University (KAU) ASD data set with 98% classification accuracy (Alhaddad et al., 2012). However, their algorithm is validated on single data set with limited subjects. They have also utilized a large number of electrodes.

### 2.2. Emotions Classification

Emotion recognition using EEG is quite complex, as it requires a careful selection of related electrode locations, suitable features, and the classification algorithm (Alturki et al., 2021). The intermixing of positive and negative emotions due to variable valence and arousal thresholds, further adds to the challenges related to the quantization of emotions (Hu et al., 2019). The data set collection is also a substantial challenge for the data quality and time involved in the induction and acquisition of participants and EEG data, respectively, for the emotion classification. Therefore, not many data sets are considered a benchmark for the EEG-based emotions classification (Koelstra et al., 2011; Zheng and Lu, 2015). The previous works have performed a very notable effort toward emotions classification and have achieved very good results (Gannouni et al., 2020; Alturki et al., 2021). They can be broadly categorized into software or hardware-based solutions. The hardware-based solutions include hardware accelerators and on-chip applications for emotion recognition, which have gained a lot of attention in recent years (Aslam et al., 2020; Aslam and Altaf, 2021a). These hardware applications require some special considerations when selecting ML or deep learning (DL) models:

1. The hardware realization on-chip has a significant cost associated with them. Therefore, their ML algorithms should be validated on multiple benchmark data sets and a large number of subjects to avoid over-fitting.
2. The large channel count would compromise patients' comfort and require more hardware resources. A detailed subject-wise channel importance analysis can identify the best suitable channel subset.
3. The best feature identification after extensive LSFE and FS is required.

Koelstra et al. (2011) proposed an emotions classification method using DEAP data set with 62% classification accuracy using Naive Bayes classifier. Their system utilized signal energies in different frequency bands as a feature set. Their method utilized a large number (32) of electrodes, was validated on a single data set and did not provide any LSFE analysis for suitable features and channels ranking.

Aslam and Altaf (2020) proposed a system on chip device for emotion recognition using two benchmark data sets including DEAP and SEED. The system provided 73.4% classification results using power spectral energy-based features and LSVM classifier using only eight channels. However, the channel selection was based on previous literature and detailed LSFE analysis for suitable channels and features identification is also missing in that work. In another system on chip device, Aslam and Altaf (2021a) performed negative emotions prediction using a deep neural network-based classifier with 85.5% classification accuracy using only two EEG channels. However, LSFE for suitable channel and feature identification is also missing in that work.

Fang et al. (2019) proposed a hardware-based emotions classification system using a convolutional neural network classifier. The EEG images after short-term Fourier transform were used as features for the classification algorithm. They have utilized only six EEG channels and achieved excellent classification results (85.5%). The main limitations of their system were a single data set validation and a lack of LSFE analysis for suitable channels and features identification.

Li et al. (2019) proposed an Graph regularized Extreme Learning Machine (GELM) classifier for emotions prediction with 88% classification accuracy. They utilized the power spectral density, differential entropy, and differential caudality-based features. Although their system was validated using three data sets including DEAP, SEED, and MAHNOOB-HCI. But, they also lack LSFE analysis to identify suitable channels and features. The number of electrodes (32) utilized by them is also high.

Liu et al. (2021) proposed a three-dimension convolution attention neural network (3DCANN) for emotions classification. Although they have provided excellent (97.3%) classification results. However, the number (64) of EEG channels utilized by them is quite high. They also lack multiple data sets validation, LSFE extraction, and analysis for suitable channel and feature identification.

Similarly, the other emotions classification systems also lack a complete LSFE and FS analysis to explore the best features and channels to achieve maximum classification results (Pereira et al., 2018; Chen et al., 2019).

Some questions related to the emotion classification are still unanswered and remain an important concern and source of confusion for an early researcher in emotions classification. We addressed them in this paper in a way that can be a guideline for early researchers:

1. **Choice of Data set:** An important question is related to the choice of data sets for emotions and ASD classifications. We have addressed the reasons for preference of certain data sets for an algorithm's validation, consideration as the benchmark data sets, and preference of specific data sets over others.
2. **Classification Threshold:** The procedure of quantifying or measuring emotions is another important confusion for early researchers. The process of emotional quantization and the impact of the classification threshold on the classification results is discussed in this paper.
3. **Channel Selection Analysis:** The selection of smaller subsets of the most suitable channels and the extraction of a limited number of features are targeted by many researchers. However, a subject-wise channel significance analysis in different data sets is missing in the literature. The subject-wise analysis using LSFE is performed in this work to select a subset of four channels most feasible for the emotions and ASD classification. The relationship between these channel locations and the corresponding human brain locations is also discussed in this work.
4. **Classification Analysis:** The methodology for emotions and ASD classification using EEG signals is also explained in this paper.

## 3. Emotions and ASD Classification Framework

The EEG signals are used to record and represent the real-time electrical activities of the brain. A set of electrodes are placed on the human scalp to record the electrical impulses from the individual neurons. The electrodes detect the superposition of these impulses in the form of electrical signals which can be utilized for emotion prediction and abnormality detection related to neurological disorders (Haputhanthri et al., 2019; Alturki et al., 2021; Taufique et al., 2021).

The placement locations of EEG electrodes/channels are standardized by an international protocol named the 10–20 system. Several studies have examined the relationship between different parts of the brain with ASD (Alhaddad et al., 2012; Jayawardana et al., 2019). The brain regions strongly related to the core ASD symptoms are the amygdala, basal ganglia, superior temporal sulcus, hippocampus, frontal gyrus, and fusiform gyrus (Philip et al., 2012; Ha et al., 2015). These regions relate to temporal, frontal, and central brain regions (Zotev et al., 2016). The frontal gyrus relates to the F7, F8, T3, and T4 electrodes and the fusiform gyrus plays a crucial role in social interaction.

**Figure 2:**
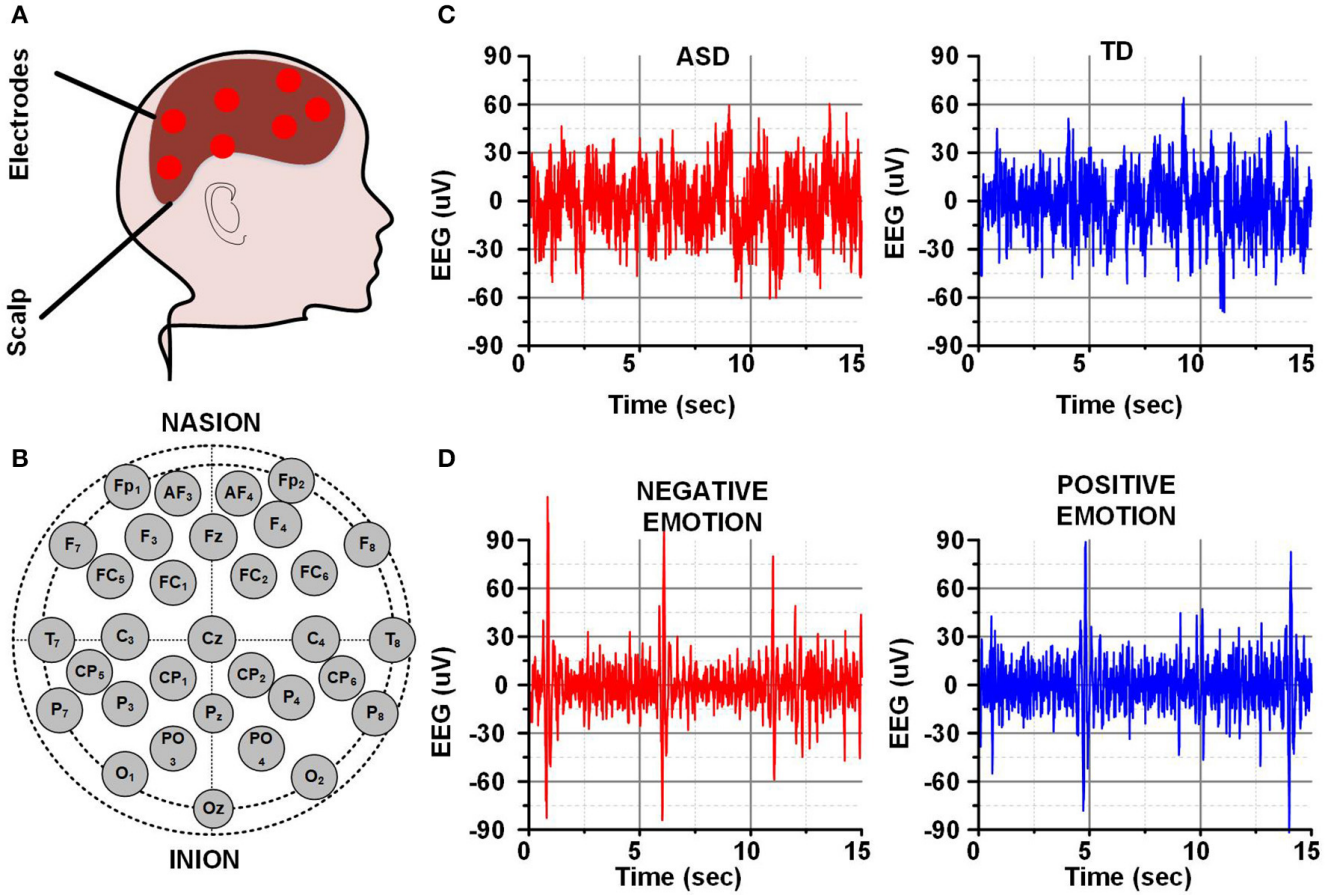
**(A)** Human brain and EEG electrodes **(B)** 10–20 EEG recording system **(C)** EEG for ASD & TD child **(D)** EEG for negative and positive emotion.

A human head with some electrodes placed on different locations on the scalp is represented in Figure 2A. The placement of different electrodes using the 10–20 placement is represented in Figure 2B. Different EEG signals are acquired from different scalp locations at a certain sampling frequency. The total amount of EEG data depends on the number of recording channels or electrodes, sampling frequency, and the recording duration. The EEG data is later utilized for brain activity analysis or neurological disorders detection as discussed earlier. The selected 15 s EEG data of a single channel for the brain activity analysis (negative and positive emotions) or neurological disorders detection (ASD and TD child) is shown in Figures 2C,D.

### 3.1. Why Machine Learning/Deep Learning?

The desired class labels are mapped to the input EEG signals by the classification process (1).


(1) $$\begin{array}{l} C=f\left(X_{11},X_{12},\ldots .,X_{MN}\right) \end{array}$$

The mathematical function *(f)* is used to map the input EEG signals (X) of n electrodes to the label (C). N and M represent the number of EEG channels and the number of recorded EEG samples for a single channel, respectively.

A large number of input variables and the inability to differentiate between the positive and negative classes with a naked eye observation necessitates the complex ML algorithms to perform this task. For example, an EEG signal of 1-min duration for 32 electrodes recorded at 128 Hz sampling frequency would have 7,680 EEG samples per channel, which becomes ≈0.25 million input samples to predict the output label using 32 channels. An ML algorithm utilizes these input EEG signals to predict the label using suitable signal preprocessing, channel selection, feature extraction, FS, feature normalization, and classification algorithms.

### 3.2. Feature Extraction and Channel Selection

The recorded EEG signals are contaminated by noise and other artifacts including eye movements, eye blinks, muscle activities, and chewing (Jiang et al., 2019). The complete set of EEG signals also contains a very large amount of data due to the large number (≥16) of channels. The presence of noise and the size of data makes it difficult for the ML classification algorithms to accurately map the input (EEG signals) to the output (label). Therefore, it is desired to remove the noise and other artifacts to identify the most relevant subset of the information required for the classification.

Channel selection is the process to identify the most suitable subset of channels out of the total number of available channels. The feature extraction process is to identify some variables or formulas instead of raw EEG signals for the classification. The EEG data of 14 to 64 channels were provided by the data set. The channel selection process selects a subset of the 4 most suitable channels. The feature extraction process calculates 32 features from these channels and the FS process selects the best 8 out of these 32 features.

The channel and FS are iterative processes and one of the most challenging procedures in ML classification. There is no fixed method to identify the best suitable features and channels for a time series EEG classification problem. Different features have to be experimented by the researchers to find the best suitable features and corresponding channels. The channel and FS are not only required for the accurate prediction or classification of the label. But they also directly affect the hardware resources for hardware accelerators or on-chip systems (Aslam et al., 2020). Generally, there are two approaches used for the channel and FS process a) self-experimentation using previous literature and domain knowledge, and b) LSFE. The LSFE techniques calculate a huge number of features from the input data, which are later on analyzed to filter the best suitable features.

The self-experimentation approach is usually considerably limited and analyzes a significantly smaller number of features as compared to the LSFE method. To the best of our knowledge, there is no previous work that has utilized the LSFE to identify the most optimal channels and features for emotions and ASD classification. Therefore, LSFE to identify the most suitable channels and features for emotions and ASD classification using DEAP, SEED, DREAMER, Old Dominion University (ODU), and KAU data set was utilized.

### 3.3. Emotions Classification Data Sets

A wide range of medically verified and annotated data sets by qualified neurologists is required to ensure the robustness of an emotions classification system (Gonzalez et al., 2021). Unfortunately, unlike the data sets for chronic neurological disorders like epilepsy, ASD, and Parkinson's disease, no medically verified and annotated data sets for emotions classification are available (Gonzalez et al., 2021). Emotions are internal feelings of a person in response to a certain event and measuring or quantifying someone's emotions is a big challenge. The unavailability of a medically established framework for the emotion's quantization can be the primary reason for the absence of medically verified and annotated emotions classification data sets (Gonzalez et al., 2021).

Emotion measurement is generally performed by self-assessment of emotions after stimulating certain emotions using audio or video stimuli. The Self-Assessment Manikin (SAM) technique scales the emotions using valence, arousal, and dominance scales (Towle et al., 1993). Valence and arousal relate to Russel's valence-arousal scale, most widely used in the emotion classification data sets (Russell, 1980). However, some data sets have also included additional dimensions of dominance, liking, and familiarity (Koelstra et al., 2011). Valence describes the positivity or negativity of emotion while arousal defines the strength of that positivity or negativity. Dominance is used to measure the sense of authority during an emotion.

The data set selection is a very critical choice for the design and verification of an emotions classification system. A researcher may claim 100% accurate classification results on a self-collected data set of a few subjects. However, the system would fail to generalize on other data sets. Therefore, we have tried to identify the data sets which are being used by the top international scientific research publication forums related to biomedical systems and healthcare. DEAP, SEED, and DREAMER are the three most popular and widely used data sets for emotions classification using physiological (EEG, ECG, EMG, EOG, etc.) signals (Koelstra et al., 2011; Zheng and Lu, 2015; Katsigiannis and Ramzan, 2017).

Careful literature analysis depicts that DEAP, SEED, and DREAMER data sets are most frequently (≈70%) utilized in the last 5 years. Therefore, we have utilized all three data sets (DEAP, SEED, and DREAMER) to analyze the human emotions classification using EEG signals (Koelstra et al., 2011; Zheng and Lu, 2015).

#### 3.3.1. DEAP Data Set

DEAP data set provides the SAM-based emotions classification for 32 participants or subjects after eliciting 40 different emotions through different video stimuli. The emotions are measured using valence, arousal, and dominance. In addition, liking and familiarity are secondary indicators used to represent the subject's previous information about the video stimulus for emotion elicitation and the participant's personal fondness of the stimulus.

**Figure 3:**
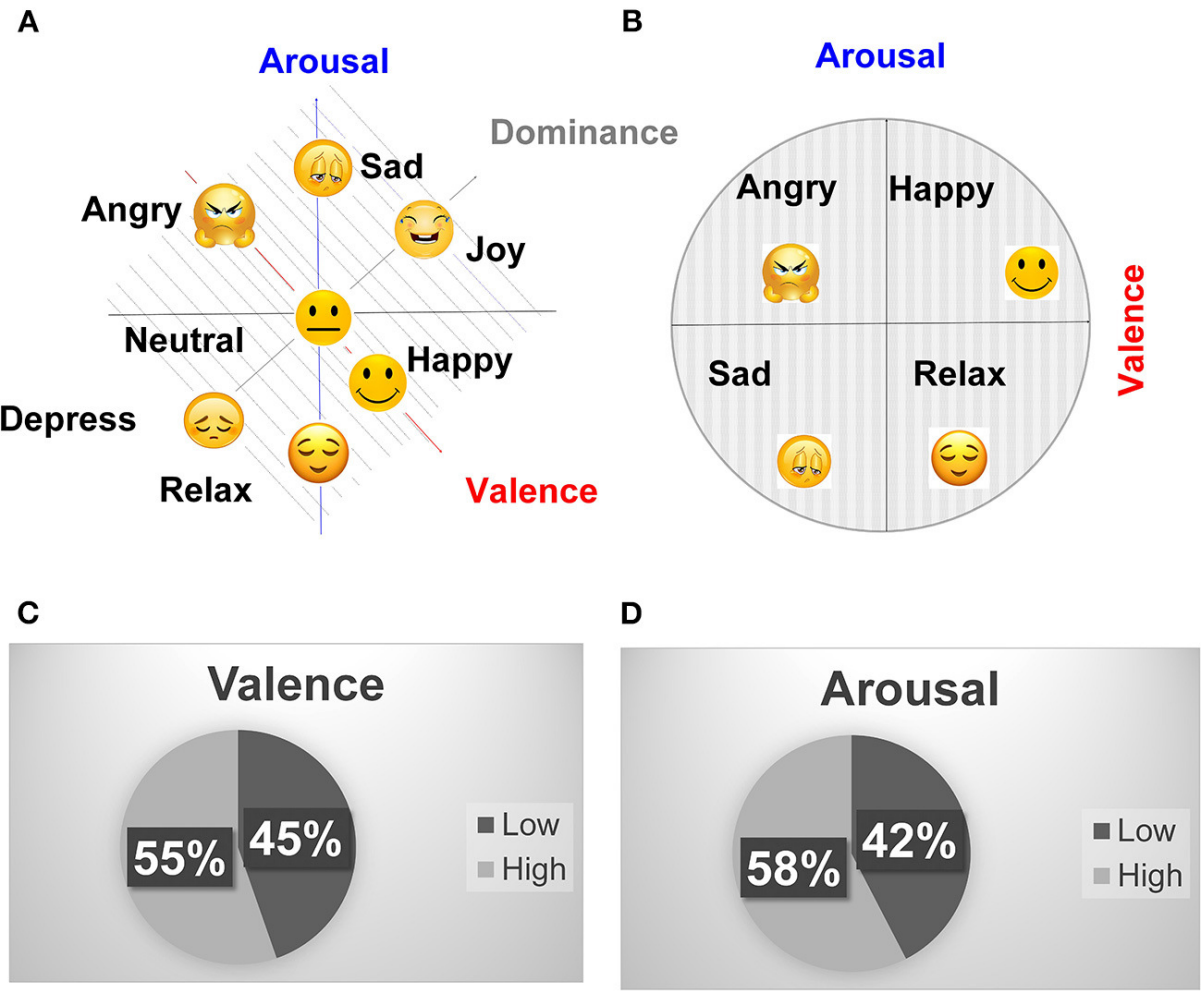
**(A)** Mapping of different emotions on valence, arousal, dominance (3-D) and, **(B)** valence arousal (2-D) scale, **(C)** DEAP valence binary classification statistics, and **(D)** DEAP arousal binary classification statistics.

Different emotions measured using the 3-D scale (valence, arousal, and dominance) and 2-D scale (valence, arousal) are depicted in Figure 3 (Gannouni et al., 2020). The happy and joy emotions are differentiated by different dominance values. Both emotions have similar valence (positive) and arousal (positive). But they have different dominance values. The joy emotion has a higher dominance than happiness. Similarly, the angry and depressed emotions have similar valence (negative) and arousal (positive), but different dominance. The angry emotion has higher dominance than the depressed emotion. Figure 3A shows the neutral, happy, joy, sad, depressed, angry, and relaxed emotions mapped using the valence, arousal, and dominance scale. It becomes hard to label a large set of emotions on the 3-D scale (valence, arousal, and dominance) (Gannouni et al., 2020). Therefore, Russel's 2-D scale (valence, arousal) is most popularly used to quantify emotions (Russell, 1980). Figure 3B shows the different emotions mapped using the 2-dimensional valence-arousal scale. It can be observed that the (angry, happy) and (sad, relax) emotions are differentiated by opposite valences and have similar arousal. Similarly, the (angry, sad) and (happy, relax) are differentiated by similar valence and opposite arousal. Since our primary focus is to differentiate between positive and negative emotions. Therefore, the analysis for valence classification was mainly focused. However, the classification results for the 2nd dimension (arousal) are also summarized.

Figures 3C,D shows the percentage of positive and negative valence and arousal classes for binary classification, respectively, using a classification threshold of 5. The valence and arousal values lesser than or equal to 5 are labeled as negative, and those greater than 5 are labeled as positive. It can be observed that the positive and negative classes are evenly balanced in the DEAP data set with the classification threshold of 5. The threshold of 3 causes the valence and arousal classes to become unevenly balanced (Positive: valence: 83.12%, arousal: 82.97% Negative: valence: 16.88%, arousal: 17.03%). The precision and recall parameters are more important than the classification accuracy for the unbalanced data sets. The balanced or unbalanced distribution of the data set is decided by the classification threshold. Therefore, for classification thresholds causing unbalanced distribution, the precision and recall should be reported. However, the classification thresholds, precision, and recall are not mentioned by a majority of the previous works, and only classification accuracies are reported. The gender statistics of the data set include 53% males and 47% female participants. It can be observed that the data set is evenly balanced between male and female genders. The data set also includes participants from all age ranges. However, most of the participants (75%) are in their twenties (20–30).

#### 3.3.2. SEED and DREAMER Data Sets

SEED and DREAMER are the other most popularly used data sets for emotions classification (Zheng and Lu, 2015; Katsigiannis and Ramzan, 2017). SEED data set provides the data for the emotions classification of 15 participants using 62 EEG electrodes (Zheng and Lu, 2015). The data set uses the valence scale only to classify an emotion as positive, negative, or neutral. The emotional classification of each subject is provided for 15 different emotional stimuli. The participants of the data set include 7 males and 8 females. The data set is evenly balanced between both genders. The average age of a participant in the SEED data set is ≈23 years. However, the age of each participant is not reported by them.

An evenly balanced distribution of the positive, neutral, and negative labels is provided in the SEED data set (33% each). Since we are performing binary classification of valence. Therefore, we discarded the neutral labels and utilized the positive and negative labels only. There is an equal percentage (50%) of positive and negative classes in the data set for binary classification of valence.

DREAMER data set provides the EEG and ECG data of 23 participants. The EEG signals are recorded using 14 electrodes through an emotive EPOC headset (Katsigiannis and Ramzan, 2017). It provides the labels of valence, arousal, and dominance for the emotion's classification. We have utilized the labels of valence and arousal only from this data set. The DREAMER data set is unevenly balanced between both genders. The valence and arousal labels in the DREAMER data set are scaled between 1 and 5. The valence and arousal values lesser or equal to 3 are labeled as low, and those greater than 3 are labeled as high. It can be observed that the binary classes of arousal are evenly balanced using a threshold of 3. However, the valence classes are unevenly balanced in the data set.

### 3.4. ASD Classification Data Sets

There are very few ASD classification data sets compared to other problems like emotions classification, epilepsy, Alzheimer. The primary reason for that can be the uncooperative behavior of ASD children, as they are generally very uncooperative. The uncooperative nature of ASD children is due to multiple reasons, a) difficulties to understand instructions, b) social interaction, and c) communication and sensory issues. The EEG classification data sets for ASD classification records the EEG data for a certain number of Autism Diagnosis Observation Schedule, second edition (ADOS-2) confirmed ASD patients and TD children.

The ASDOS-2 is a standard ASD diagnosis method. It requires a communication score (CSC), social interaction score (SCI), imagination and creativity score (IMC), stereotyped behaviors score (STB), and their cut-off values with a certain threshold table evaluated by the neurologists. A behavioral diagnosis cycle of the patient evaluates the SCI, CSC, STB, and IMC scores of the patient. The scores are compared with the cut-off table and then the patient is labeled as ASD or TD.

The EEG-based ASD classification system is trained to predict the patient as ASD or TD using EEG signals. The predicted labels are then compared with the original labels assigned through the ADOS-2 method. There is no publicly available EEG-based ASD data set to the best of our knowledge. We have utilized the ASD data sets by Old Dominion University (ODU), USA (Jayawardana et al., 2019) and KAU, KSA (Alhaddad et al., 2012) shared with us for our research.

ODU data set (Jayawardana et al., 2019) provides the EEG data of 17 subjects including 8 ASD and 9 TD subjects. The subjects include 10 males and 7 females. The males include 6 ASD and 2 TD subjects. The females include 2 ASD and 5 TD subjects. The ODU data set has also provided the ADOS-2 scores of each patient on which they were labeled as ASD or TD. The ASD patients have a higher ADOS-2 score than the TD subjects. The ODU data set has recorded the EEG signals of all subjects using 32 electrodes. However, in our analysis, we observed that the EEG signals for only 14 electrodes (F7, F3, Fz, F8, FC1, FC2, FC6, T9, T7, C3, T10, CP5, CP2, P7, and P3) were available across all subjects. The EEG data of the remaining electrodes were either missing in some subjects or too noisy to be included in the analysis. Therefore, we have focused only on these 14 channels in this work.

KAU data set provides the EEG data of 12 children including 8 ASD children and 4 TD children (Alhaddad et al., 2012). The data set does not provide the ADOS-2 scores of the patients. The ASD children include 5 boys and 3 girls whereas the TD children include 4 boys. All the participants were aged between 10 and 11 years. They have recorded the EEG data using 16 channels (FP1, FP2, F7, F3, Fz, F4, F8, T3, C4, Cz, C3, T5, Pz, O1, Oz, and O2).

## 4. Large Scale Feature Extraction And Classification Methodology

The feature extraction process for the time series classification problems, including emotions recognition and ASD prediction, is hectic (Fulcher, 2017). It requires the analysis of a large combination of features using previous domain knowledge and experimentation with different features. Some LSFE packages including TSFRESH, TSFEL, HCTSA, etc using MATLAB or python implementations are proposed by different researchers (Christ et al., 2018; Barandas et al., 2020). A large set of features using the time series EEG data is calculated by these packages. These features can be passed through different FS methods, including selecting k best (SKB), sequential forward search (SFS), etc. to select the best optimal feature subset. The FS methods utilize different learning algorithms to find the best subset of features from the large feature set acquired through the LSFE method.

**Figure 4:**
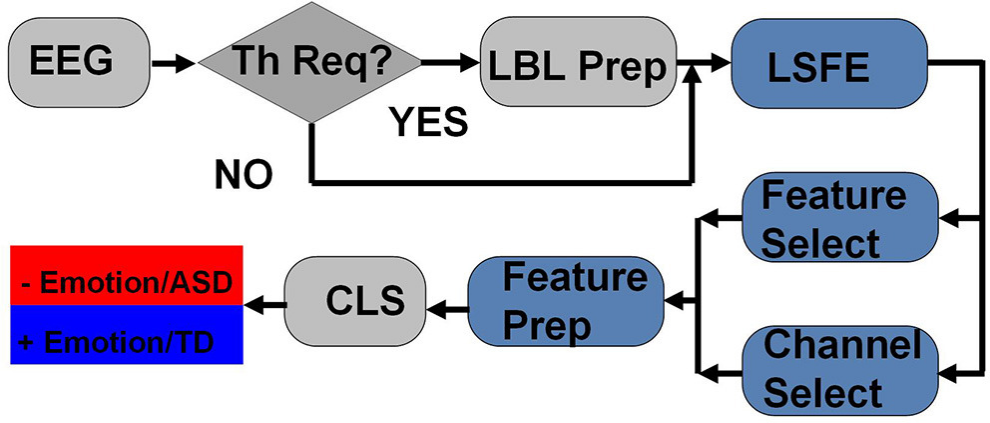
LSFE and classification methodology.

The block diagram for the emotions and ASD prediction methodology using LSFE is depicted in Figure 4. The complete set of prepossessed EEG data is passed through the threshold process for label creation if required. If the data set has already provided the binary labels for positive and negative emotions or ASD/TD classification, then the threshold process is not required. The EEG data and labels are forwarded to the LSFE method (TSFRESH etc.) for LSFE analysis. A large set of features is calculated by the LSFE process. The LSFE features are forwarded to the channel and feature selection methods. The small subset of features after suitable channel and feature selection is used for the preparation of the feature set. The final feature set after feature preparation is provided to the classification method (CLS) for positive/negative emotions or ASD/TD classification.

The TSFRESH package for the LSFE of emotions classification and ASD prediction was utilized in this work. The TSFRESH package provides 63-time series characterization methods and calculates a total of 794 features for each EEG channel. It can be used for both univariate and multivariate time series and can handle variable-length time series. Some of the features used in TSFRESH include mean absolute energy, absolute maximum, number of peaks, quantile value, and zero crossings. An exhausting list of features extracted in TSFRESH is listed in Christ (2016).

The large-scale feature matrix is then forwarded to the FS method to find the best subset of features. The SKB and SFS methods are used in this work (Pedregosa et al., 2011). SKB is an implementation of a filter FS method where it eliminates all excluding the highest-scoring features. The number of selected features is controlled by the k value. The SKB uses a linear method to select features that contain information about the target variable using statistical tests such as ANOVA, Fisher score, and Chi-Squared (Gómez-Raḿırez et al., 2020). The selection of important features was based on the ANOVA test with *k* = 8. SFS is an iterative wrapper-based method (Gómez-Raḿırez et al., 2020). It starts with an empty set and adds features to form a feature subset. Those selected features give the highest value for the objective function. The objective function is defined by a perceptron in our case. The FS is a back-and-forth learning process through the relevant learning algorithm. The FS methods work to reduce the size of the feature matrix. They reduce over-fitting by excluding redundant features, improving the classification results, and decreasing the classifier's training time. LSFE and FS are performed for the selected data sets. The results of valences in emotions classification data sets are presented in detail, whereas the results of arousal classification are briefly summarized in this paper.

### 4.1. Emotions Classification LSFE

The DEAP, SEED, and DREAMER data sets include the data of 32, 62, and 14 EEG channels, respectively. Several previous works have identified different smaller pools of most suitable channels for emotion classification with customized feature sets (Koelstra et al., 2011; Pereira et al., 2018). However, a detailed analysis of all EEG channels was not performed to show the significance of their channel subsets. The utilization of different channels using LSFE to identify the best suitable channel subset was analyzed in this work.

**Figure 5:**
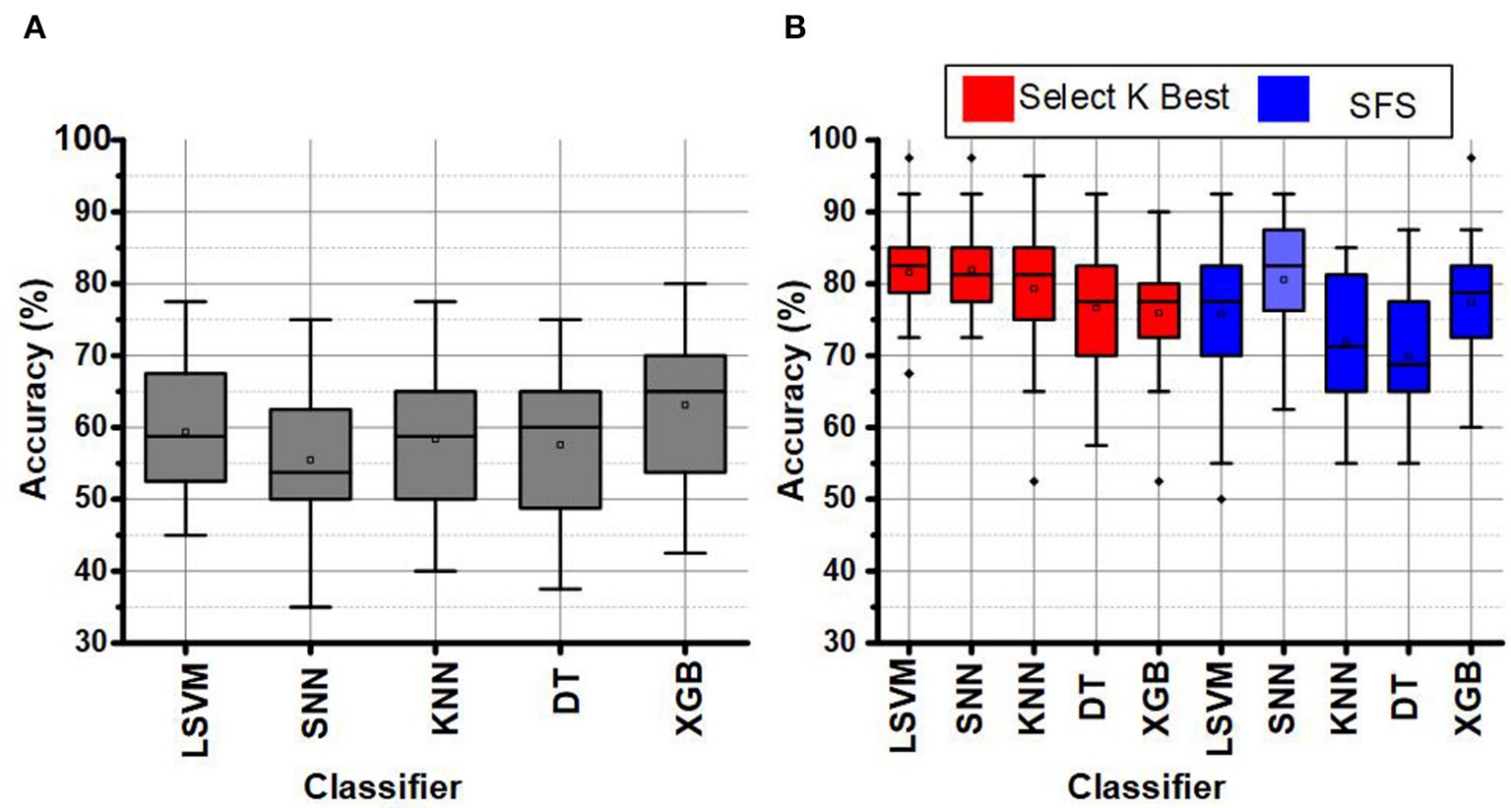
**(A)** LSFE classification of EEG signals, and **(B)** FS using SKB and SFS for DEAP data set.

The LSFE matrix calculated a considerable number (≈25 K) of features for the DEAP data set using 32 EEG channels. The classification performance using the LSFE matrix was not satisfactory (≤ 65%). The primary reason for the low classification results using LSFE matrix with all EEG channels was over-fitting and redundant features. The SKB and SFS methods significantly improved the classification results (~85%) for emotions classification. Figure 5 represents the classification performance of LSVM, SNN, KNN, DT, and XGB classifiers using the LSFE matrix before and after FS. Figure 5A represents the classification results using the LSFE matrix. Figure 5B represents a box chart of the subject-wise classification results after FS for all subjects in the DEAP data set. The red and blue colors in Figure 5B indicate the classification results of SKB and SFS methods, respectively. It can be observed that the classification results were significantly improved.

**Figure 6:**
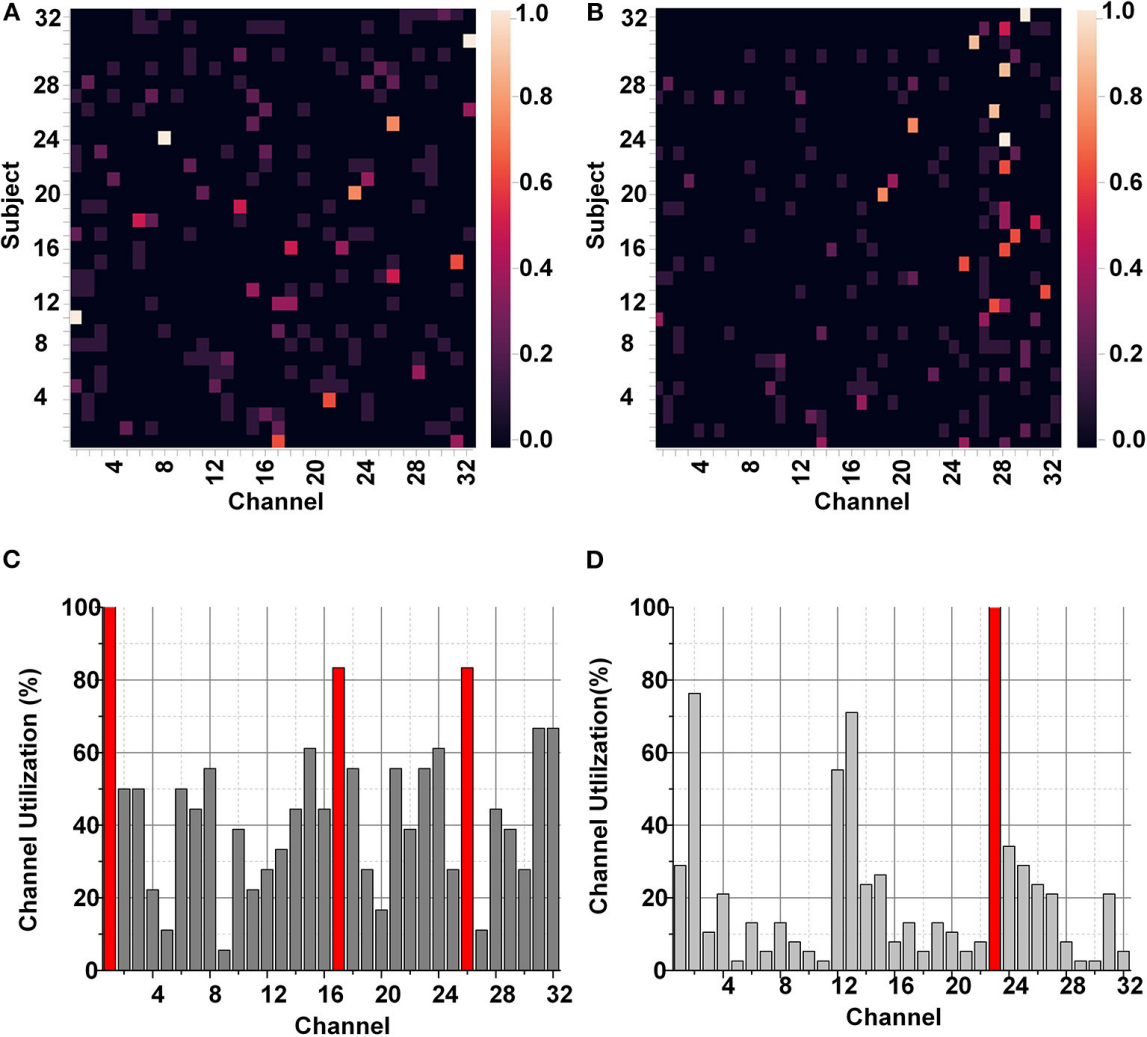
**(A)** Heat map for channel selection using SKB, **(B)** Heat map for channel selection using SFS, **(C)** Bar graph for channel selection using SKB, and **(D)** Bar graph for channel selection using SFS for DEAP data set.

The identification of a smaller subset of suitable channels was performed using the SKB and SFS methods. These FS methods were tuned to utilize a set of 8 best suitable features for the classification. As a result, the FS methods improve the classification performance and significantly impact (>3k times) the hardware resources for hardware accelerators and on-chip applications. Figure 6 represents the utilization of each EEG channel using the SKB and SFS methods. The heat maps in Figures 6A,B represent the channel importance using SKB and SFS, respectively. It can be observed in Figure 6A that channel number 1 (FP1) is utilized for all features (8/8) using the SKB method for subject number 11.

The maximum (8/8) and minimum (0/8) utilizations of the channel are reflected by white and black colors in the heat map, respectively. However, it was difficult to completely analyze the utilization of each channel for each subject using the heat maps. Therefore, we have presented the accumulated utilization percentage of each channel using SKB and SFS in Figures 6C,D, respectively. These bar graphs show the accumulated utilization of each channel for all subjects in the DEAP data set. It can be observed that channel numbers 1 (FP1),17 (FP2), 23 (FC2), and 26 (T8) were most suitable for the emotion classification. The T8 channel was also observed to be among the eight-channel pool in our previous work. This channel subset relates to the amygdala region of the brain (Tong et al., 2019). The amygdala region of the brain is closely linked to human emotions (Suhaimi et al., 2020). After LSFE, the most significant features identified using these FS methods include autocorrelation, Fourier transform coefficients, signal energy, continuous wavelet transform coefficients, change quantiles, and aggregated least square regression.

**Figure 7:**
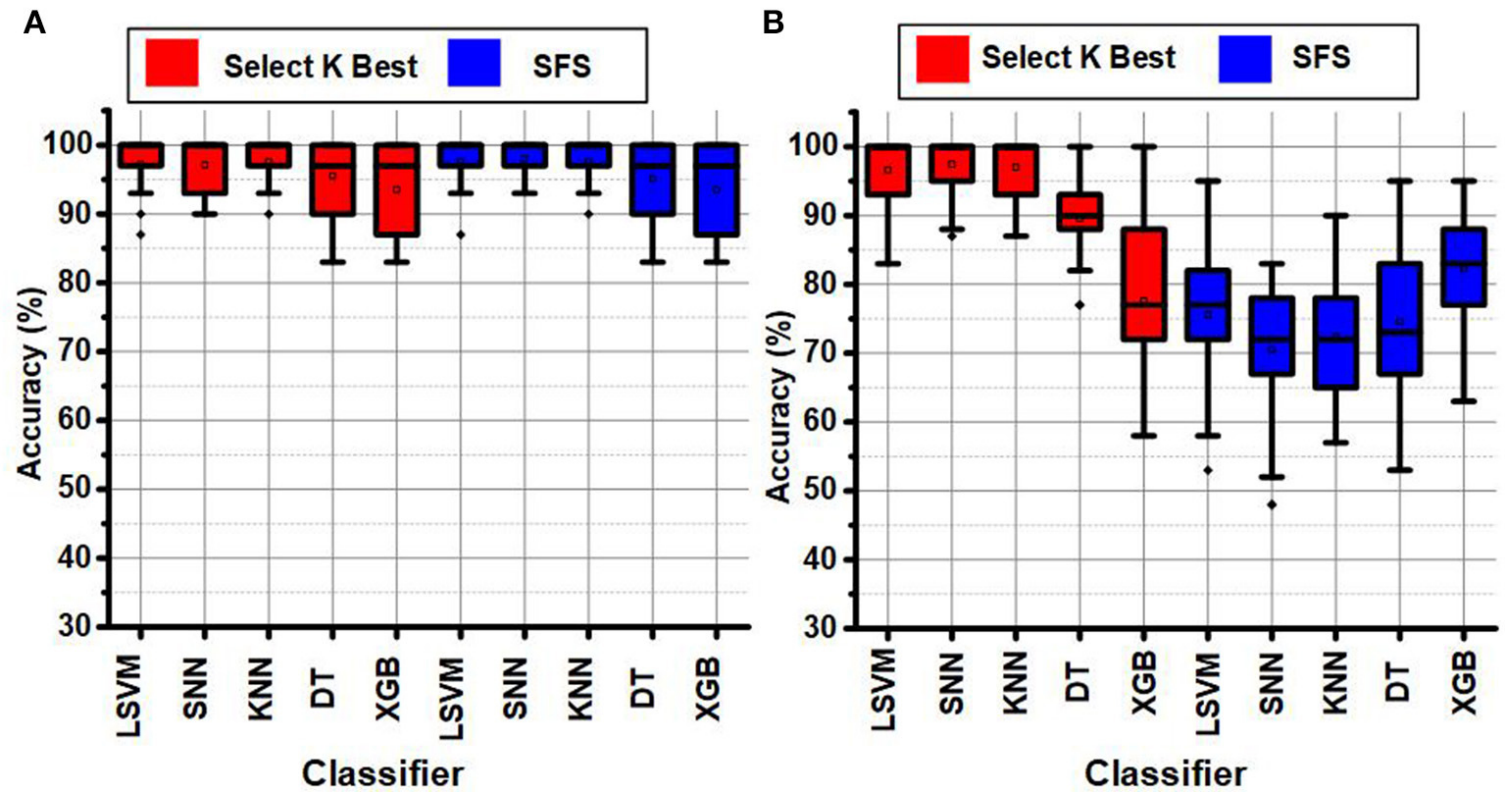
Classification results after feature selection for **(A)** SEED, and **(B)** DREAMER data set.

The LSFE feature extraction and FS methods improved the classification results for SEED and DREAMER data set significantly compared to the DEAP data set. Figure 7 presents the classification results of SEED and DREAMER after FS and LSFE using TSFRESH. Figure 7A shows the classification results for the SEED data set, and Figure 7B shows the classification results for the DREAMER data set. The red and blue colors represent the SKB and SFS methods, respectively. Both FS techniques provided excellent classification results (~98%) for SEED and DREAMER data sets.

**Figure 8:**
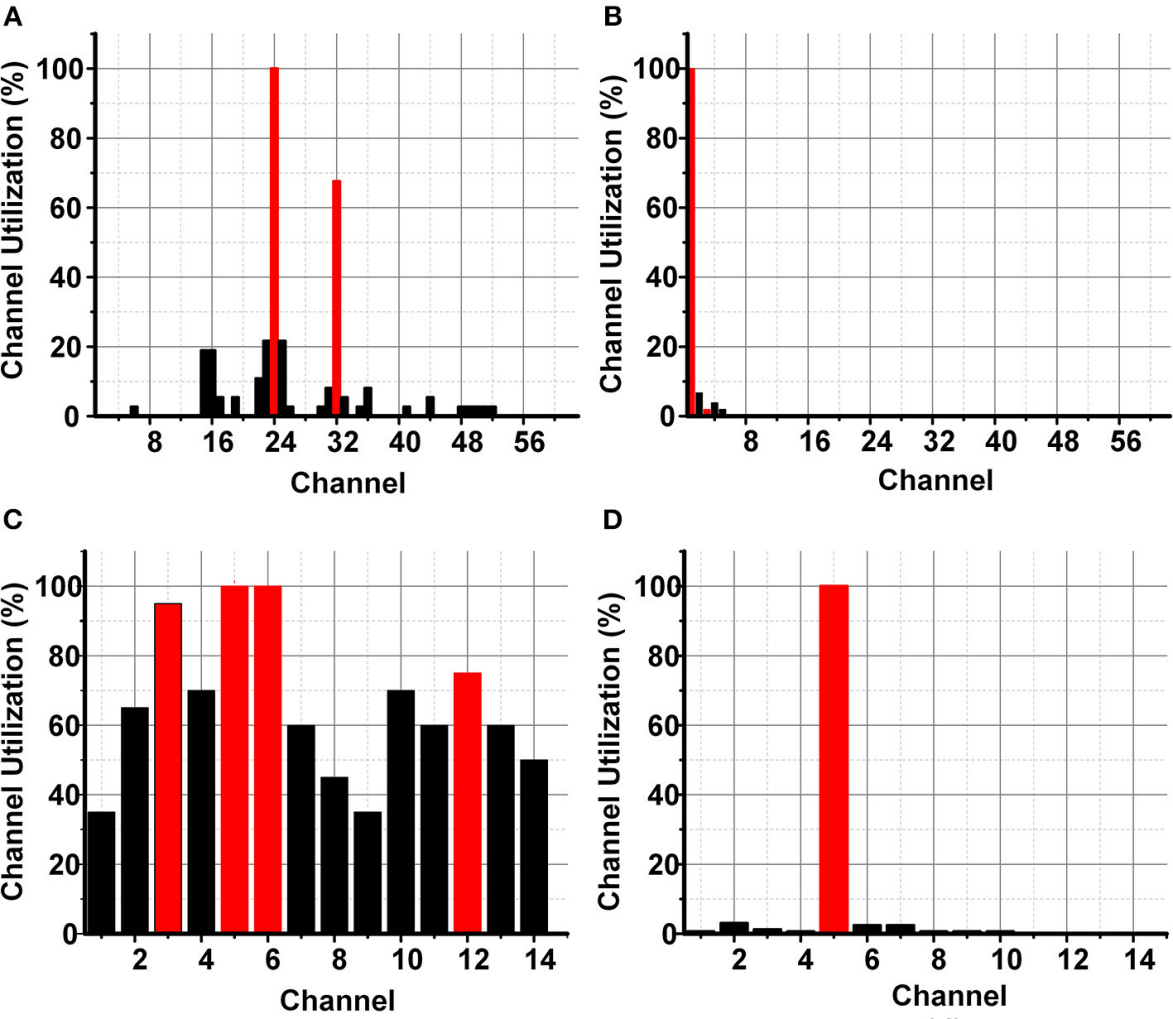
Channel importance for SEED data set using **(A)** SKB, and **(B)** SFS, and DREAMER data set using **(C)** SKB and **(D)** SFS.

The identification of a smaller subset of suitable channels (4 channels) for the SEED and DREAMER data sets would reduce the hardware resources to ≈12.5 and ≈3.5 k times, respectively. Figure 8 shows accumulated channel utilization for SEED and DREAMER using SKB and SFS methods. Figures 8A,B represent the channel importance for the SEED data set using SKB and SFS, respectively, whereas Figures 8C,D represent the same for the DREAMER data set.

The channel utilization analysis suggested that the channel numbers 24 (T7), 32 (T8), 1 (FP1) were significantly important in the SEED data set. It can be observed from Figures 8A,B that only three EEG channels have significantly important utilization. The channel combination (T7, T8) confirmed our previous observations of the significance of temporal channels and asymmetric electrodes combination for emotions classification (Aslam et al., 2020). The channel number 3 (FP2) was included as the fourth channel as a 4-channel subset was targeted. This channel selection was used to exploit the benefit of asymmetric electrodes combination (Aslam et al., 2020). The channel combination (T7, T8, FP1, FP2) further improved the classification results as discussed later in this section. Similarly, channels 3 (F3), 5 (FC5), 6 (T7), and 12 (F4) were observed to be significantly important for the emotion classification in the DREAMER data set.

### 4.2. ASD Classification LSFE

The ODU ASD classification data set provides the EEG signals of 14 channels for all subjects as explained earlier in the data set section (Jayawardana et al., 2019). The LSFE matrix provided ≈11 k features for the ODU data set. The LSFE matrix provided a maximum accuracy of 83% for the ASD classification (KNN classifier).

**Figure 9:**
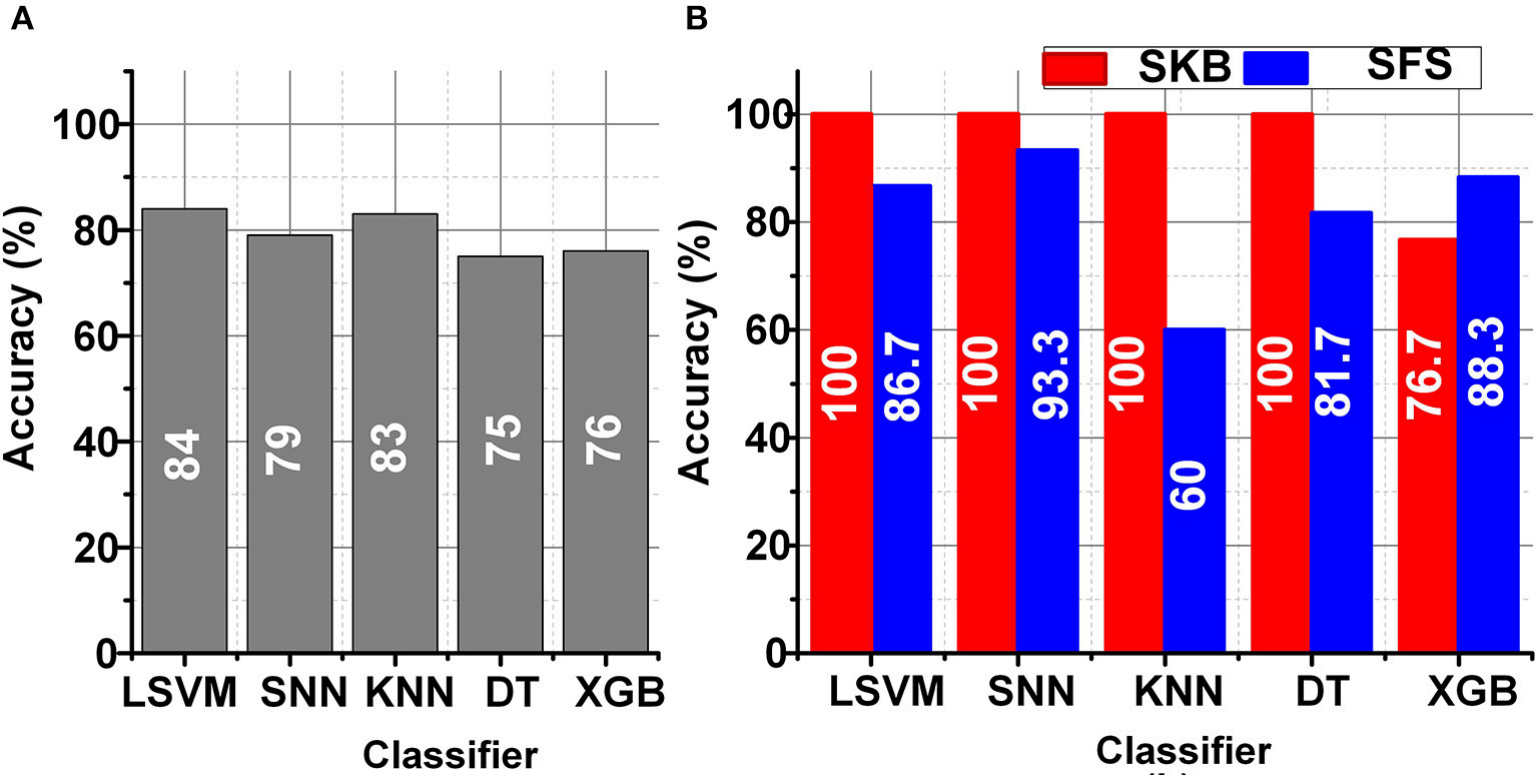
Classification results for ASD classification **(A)** before FS **(B)** after FS using ODU data set.

Figures 9A,B show the classification results for ASD classification for the ODU data set using LSVM, SNN, KNN, DT, and XGB classifiers before and after FS, respectively. The SKB and SFS methods were used to extract the best 8 features from the LSFE matrix. The SKB methods provided excellent classification results (100%) whereas the SFS method provided the maximum classification accuracy of 93%. The FS methods would reduce the hardware complexity ≥ by 1.4 k times for the hardware accelerators and on-chip applications.

**Figure 10:**
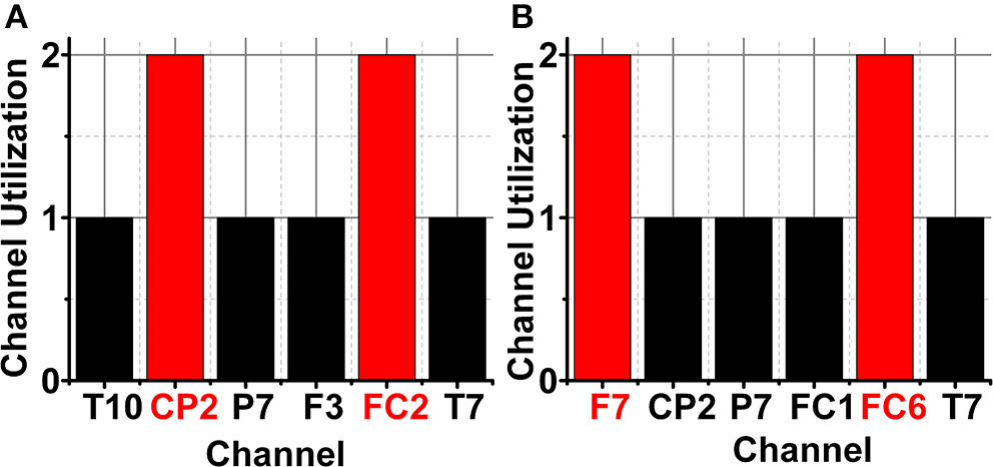
ODU data set channel importance for ASD classification **(A)** LSFE matrix **(B)** SKB and SFS.

The identification of a smaller subset of suitable channels (4 channels) for ASD classification would also reduce the discomfort for ASD children for wearable on-chip applications. Figure 10 shows the channel importance of different channels for ASD classification with SKB and SFS methods using the ODU data set. The subset of 8 channels using SKB and SFS are shown in Figures 10A,B, respectively. It can be observed that the channel locations CP2, FC2, F7, and FC6 have the maximum utilization among the 8 feature subsets. Therefore, these channels were identified to be most suitable for ASD classification using the ODU data set.

The KAU data set provides the EEG signals of 16 channels for 12 subjects as explained earlier in the data set section. The LSFE matrix provided ≈12.5 k features for the KAU data set. The LSFE matrix provided a maximum classification accuracy of 77% for the ASD classification using the SNN classifier. Both the FS methods provided excellent classification results (100%) after FS. The FS methods would also significantly impact the hardware complexity ≥1.6 k times for the hardware accelerators and on-chip applications.

Similarly, the channel locations F3, T5, O1, and O2 have the maximum utilization among the 8 feature subsets in the KAU data set. Therefore, these channels were identified to be most suitable for ASD classification using the KAU data set. The most suitable features for ASD classification include Fourier transform coefficients, auto-correlation, and mean absolute energy.

### 4.3. Channel and Feature Subset Settlement

The settlement or consensus of the identified channel and feature subset is required to conclude the suitable channel and feature subset for emotions classification. The primary reason for this is the subject-wise classification and dependence of the channel and feature subsets. The channel and feature combination proposed in this work provided the best classification results. However, due to the subject-wise dependence on emotions classification, the selected subject of channels may not correspond to the FS of many subjects.

**Figure 11:**
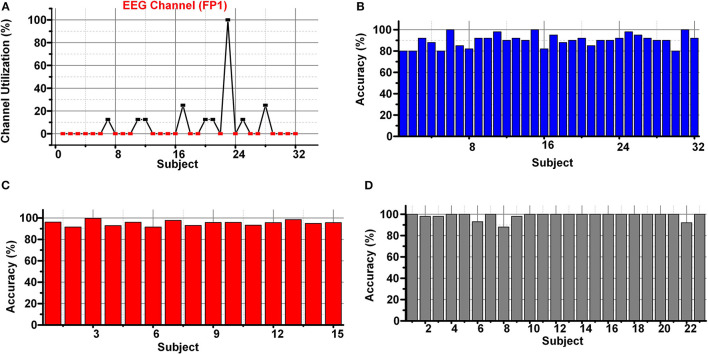
**(A)** Channel importance of FP1channel in DEAP **(B)** classification results using brute force approach in DEAP **(C)** SEED **(D)** DREAMER data set.

Figure 11A shows the channel utilization percentage of the best EEG channel (FP1) in the DEAP data set using SKB. The best feature subset using SKB does not contain any feature corresponding to the FP1 channel for multiple subjects. Therefore, it is impractical to have a subject-specific channel selection. But the classification results were degraded (≈10%) using the selected subset of 4 channels.

The primary challenge of the research for emotions classification using EEG and peripheral signals is to discover a subject independent channel and feature subset feasible for the classification. Therefore, the LSFE and FS process to select the best 32 features using the subsets of 4 EEG channels using DEAP, SEED, and DREAMER data sets was repeated. The selected features include auto-correlation, change quantiles, entropy, signal energy, aggregated linear trend, welch density, Fourier entropy, and auto-correlation. A brute force approach was applied to select the best possible subset of features from all possible (≈10 million) 8 feature subsets. The classification results from the brute force approach using DEAP, SEED, and DREAMER data sets are shown in Figures 11B–D. The average classification results of 90.1, 95.22, and 98.56% were achieved using DEAP, SEED, and DREAMER data sets for valence classification using the LSVM classifier.

The LSVM classifier was selected based on the classification results and the classifier's complexity. A subset of eight features is forwarded to the classifier for positive or negative emotion/ASD classification. The dot product of the feature vector is calculated with the classifier weights (2). A positive or negative class is assigned based on the comparison of the dot product with a constant K. The LSVM process assigns the positive or negative class assignment to a feature based on the dot product.


(2) $$\begin{array}{l} C=F.W\geq K \end{array}$$

The complexity of the LSVM classifier is O(n) where n is the number of input dimensions (Abdiansah and Wardoyo, 2015). The number of input dimensions is dependent on the number of features. The LSVM classifier is chosen due to the classification results and lower complexity than deep neural networks (DNN) or convolutional neural networks (CNN). The DNN or CNN with the same or more input dimensions than an LSVM would always have a higher complexity than the LSVM due to activation functions. For e.g., a shallow neural network with eight input nodes and one output node with a sigmoid activation function would have more computational complexity than an LSVM with eight input features. We have therefore preferred the LSVM classifier.

## 5. Results

Emotions and ASD classification and its analysis using DEAP, SEED, DREMAER, ODU, and KAU data sets were performed in this study. A total of 99 subjects were classified and analyzed. The emotions classification included positive or negative valence and arousal classification for the DEAP data set. The SEED and DREAMER data sets were utilized for the positive or negative valence classification only. The DEAP data set provided the valence and arousal values from 1 (minimum) to 9(maximum). A classification threshold of 5 was used in the data set to mark the labels as positive or negative. The SEED data set does not require a classification threshold as the positive or negative valence labels were provided by the data set. The valence values from 1 to 5 were provided in the DREAMER data set. A classification threshold of 3 was used to mark the labels as positive or negative valence. The ASD classification data sets (ODU and KAU) provided the subjects labeled as ASD (positive) or TD (negative).

The emotions and ASD classification was performed using LSVM, DNN, KNN, DT, and XGB classifiers. The LSVM classifier provided the overall best-case classification results. A subset of only 4 EEG channels was utilized for the classification. The classification result and the four EEG channels subset are summarized in Table 1.

**Table 1:** Classification results of emotions and ASD data sets using EEG signals.

**Data set**	**Parameter**	**Channels**	**Accuracy(%)**
DEAP	Valence	FP1, FP2, FC2, T8	90.1
	Arousal		93.5
SEED	Valence	T7, T8, FP1, FP2	95.2
DREAMER	Valence	F3,F4,FC5,T7	98.6
ODU	ASD	CP2, FC2, F7, FC6	100
KAU	ASD	F3, T5, O1, O2	95.5

The classification accuracies of 90.1 and 93.5% were achieved for the valence and arousal classification, respectively, in the DEAP data set. The SEED data set was classified for valence classification with 95.2% accuracy. The DREAMER data set was classified with 98.6% classification, respectively for valence classification. The ASD classification was performed with 100 and 95.5% classification accuracy using ODU and KAU data sets, respectively.

The selected four EEG channels included FP1, FP2, FC2, and T8 channels for the DEAP data set. The SEED data set provided the best classification results using T7, T8, FP1, and FP2 channels. The selected four channels for the DREAMER data set included F3, F4, FC5, and T7 channels. The four EEG channel subsets for the ODU data set included the CP2, FC2, F7, and FC6 channels. The selected channels for the KAU data set include F3, T5, O1, and O2 channels.

**Figure 12:**
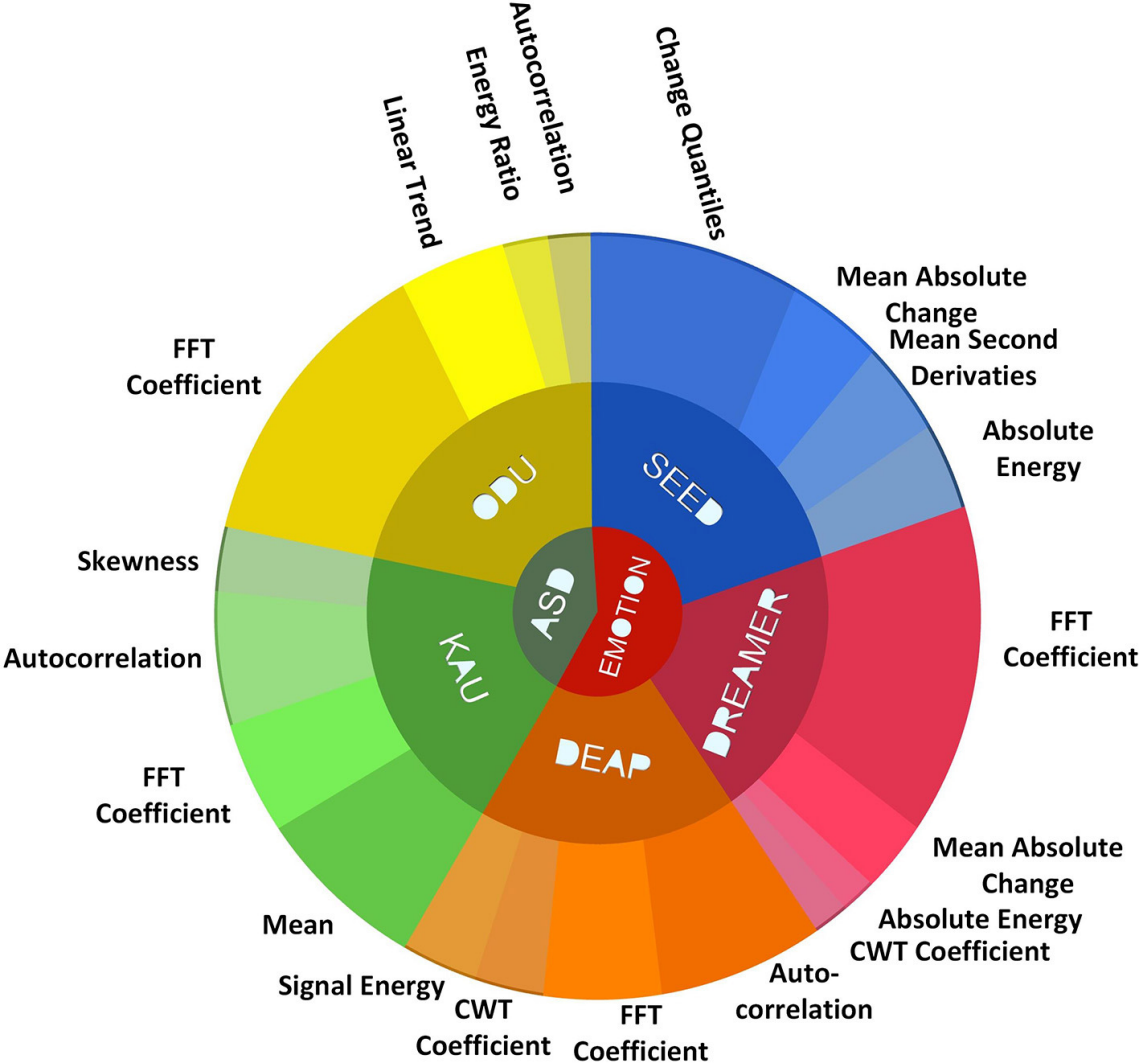
Features wheel diagram for emotions and ASD classification.

A set of eight features for the four EEG channels was used for the classification. The eight features are calculated using the brute-force approach from the set of 32 suitable features identified for emotions /ASD classification. The selected features include the fast Fourier transform coefficients, skewness, mean, signal energy, continuous wavelet transform coefficients, auto-correlation, absolute energy, mean absolute change, mean second derivative, change quantiles, energy ratio, and linear trend for emotions and ASD classification. The frequency of the selected features for emotions and ASD classification for each data set is plotted through a wheel diagram in Figure 12.

The auto-correlation, energy ratio, linear trend, fast Fourier transform coefficients, skewness and mean features were observed to be highly significant for the ASD classification. Among the selected features for ASD classification, fast Fourier transform coefficients and auto-correlation were observed to be more significant irrespective of the data set. The emotions classification using the DEAP, SEED, and DREAMER data sets provided the maximum classification results using the change quantile, mean absolute change, mean 2nd derivative, absolute energy, fast Fourier transform coefficients, auto-correlation, signal energy, and continuous wavelet transform coefficients. The fast Fourier transform coefficients, mean absolute change, continuous wavelet transform coefficients, and absolute energy was observed to be more significant across multiple data sets for emotions classification as depicted in Figure 12.

## 6. Discussion

This paper provides a framework for the researchers performing emotions and ASD classification using ML. The most frequently utilized and "benchmark-considered" data sets for the emotions and ASD classification including DEAP, SEED, DREAMER, ODU, and KAU data sets were utilized. A detailed analysis of the data sets including the number of subjects, number of classes per subject, range of label values including valence and arousal, gender and age statistics of each subject, the importance of classification threshold for emotions classification, and the percentage of the positive-negative split in each data set was described.

A brief comparison of the proposed LSVM classifier with selected features and channels with the previous state-of-the-art works using NB, GELM, 3DCANN, RNN, KNN, and CSVM classifiers for emotions and ASD classification is presented in Table 2. The classification problem (CLF Prob), Channel count (Ch. Count), best-case percentage accuracy (Acc%), number of validation data sets, and the channel ranking are mentioned in the table.

**Table 2:** Comparison with the related works.

	**TAC**	**TBIOCAS**	**TBME**	**JETCAS**	**JBHI**	**AICAS**	**ACCESS**	**ACCESS**	**CMP.BIO.MED**	**This**
	**2012**	**2020**	**2019**	**2019**	**2021**	**2021**	**2020**	**2021**	**2021**	**Work**
CLF Prob	EMT	EMT	EMT	EMT	EMT	ASD	ASD	ASD	ASD	EMT+ASD
Ch. Count	32	8	32	6	62	4	16	16	64	4
Classifier	NB	LSVM	GELM	CNN	3DCANN	LSVM	RNN	KNN	CSVM	LSVM
ACC %	62	73.4	88	83.4	97.3	85.5	99.5	98.5	96.4	98.6/100
# of Sub	32	47	74	32	15	12	12	17	54	99
Data Set (#)	1	2	3	1	1	1	1	1	1	5
Ch. Rank	X	X	X	X	X	X	X	X	X	O

*Contributions of this work are highlighted in red*.

All the previous works have focused on the emotions or ASD classification and none of them have provided a single framework for both classification problems (Koelstra et al., 2011; Fang et al., 2019; Li et al., 2019; Aslam and Altaf, 2020; Bouallegue et al., 2020; Alturki et al., 2021; Aslam et al., 2021; Baygin et al., 2021; Liu et al., 2021). This is the first study (to the best of our knowledge) that provides a framework and guideline for both emotions (EMT) and ASD classification. The significance of EEG-based emotions and ASD classification systems is highly dependent on the channel count, especially for wearable systems (Aslam and Altaf, 2019; Fang et al., 2019). The identification of a minimum (4) number of channels was a primary focus of this study. This study also provided the maximum classification results of 98.6 and 100% classification accuracies for the emotions and ASD classification. The highest number (99) of subjects were analyzed in this study, with the largest number (5) of data sets. This is the only study that provided a detailed analysis for the channel ranking to identify the best four suitable EEG channels.

**Figure 13:**
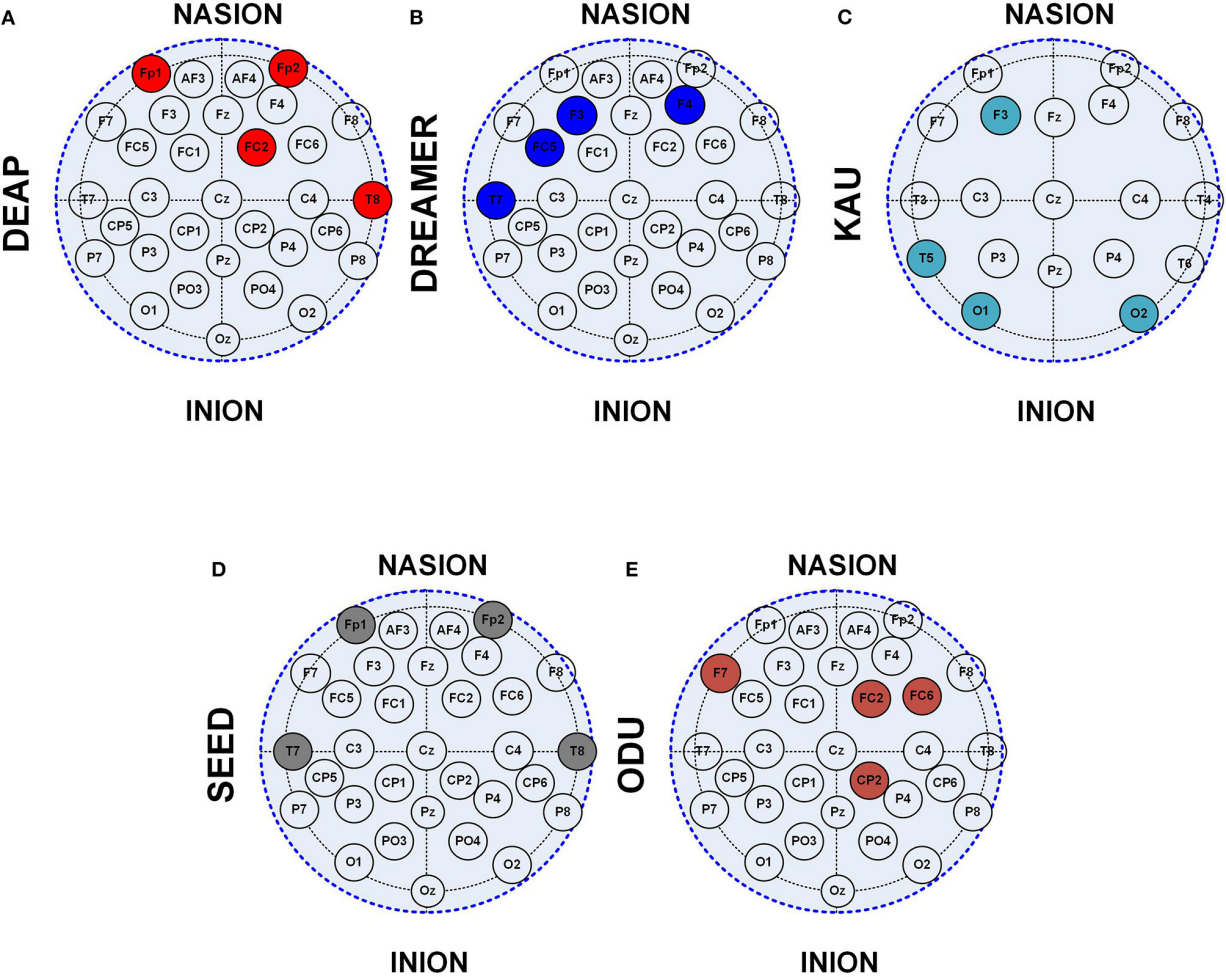
Location of 4 channel EEG subset using 10–20 system on **(A)** DEAP, **(B)** SEED, **(C)** DREAMER, **(D)** ODU, **(E)** KAU data sets.

The location of the EEG channels becomes highly significant for the hardware-based emotions and ASD prediction systems in addition to the channel count. The location of the shortlisted four channels using the 10–20 system is shown in Figure 13. It can be observed that temporal and frontal locations are highly significant for the emotions prediction, irrespective of the data set. The significance of the prefrontal (FP) region is higher than in other regions and can be observed from DEAP and SEED data set analysis through Figures 13A,D. Similarly, the fontal-central and occipital brain regions were observed to be highly significant for the ASD classification.

## 7. Conclusion

This paper has provided a detailed guideline to the early researchers for the EEG-based emotions and ASD classification. We have described the procedure to quantify and measure human emotions, along with the characterization of multiple data sets for the emotions and ASD classification. The most frequently used and "benchmark-considered" data sets among the leading researchers are used. The data sets included DEAP, SEED, DREAMER, ODU, and KAU data sets for emotions and ASD classification. The importance of the classification threshold in the emotions classification is highlighted. The balanced class distribution for positive and negative classes is changed to an unbalanced distribution for a classification threshold lower or higher than 5 in the DEAP data set. The significance of classification accuracy is decreased for the unbalanced class distribution and other classification results including precision, recall, and F1 score, become more important. Therefore, it is quite important that the classification threshold in addition to the classification results should be reported for emotions classification.

This work has also performed the ASD classification using ODU and KAU data sets. The conventional ADOS-2 method for ASD diagnosis and the ADOS-2 scores confirmation with the aid of neurologists is also detailed. The proposed method provided the highest classification results of 100% accuracy for the ODU data set and 95.5% accuracy for the KAU data set. This work has primarily focused on an extensive analysis through LSFE to identify the most suitable channels and brain areas for emotions and ASD classification. We achieved higher classification results than the state-of-the-art using a lower number of channels for emotions and ASD classification. The algorithm was validated on a maximum number of subjects and benchmark data sets. The LSVM classifier used in this work has significantly lower complexity than the other classifiers. The identified brain areas for the emotions classification included the temporal, frontal, and prefrontal regions. The identified regions for the ASD classification included the occipital and frontal-central regions. A subset of the most suitable four EEG channels was identified for each data set which is highly beneficial for the researchers targeting a minimum number of channels. The feature significance highlighted by the extensive LSFE analysis is highly important, especially for the researchers targeting hardware-based systems. The approximated hardware implementations of the highlighted features can be used to develop low-cost (area, power, and energy) hardware systems for emotions and ASD prediction.

## Data Availability Statement

Publicly available datasets were analyzed in this study. This data can be found here: https://bcmi.sjtu.edu.cn/home/seed/; https://zenodo.org/record/546113. Further inquiries should be directed to the corresponding author.

## Author Contributions

AA and NH developed, implemented, evaluated the algorithms, wrote, and revised the manuscript. HH and MA reviewed and supervised the research. All authors contributed to the article and approved the submitted version.

## Funding

This work was funded by EPSRC Industrial CASE (EP/W522168/1), Analog Neuromorphic Processing for Biosensors Grant, and Commonwealth Split Site Research Fellowship for AA. This work was also supported by the Syed Babar Ali Research Fellowship, Lahore University of Management Sciences, Pakistan for AA.

## Conflict of Interest

The authors declare that the research was conducted in the absence of any commercial or financial relationships that could be construed as a potential conflict of interest.

## Publisher's Note

All claims expressed in this article are solely those of the authors and do not necessarily represent those of their affiliated organizations, or those of the publisher, the editors and the reviewers. Any product that may be evaluated in this article, or claim that may be made by its manufacturer, is not guaranteed or endorsed by the publisher.
